# Aberrant DNA methyltransferase expression in pancreatic ductal adenocarcinoma development and progression

**DOI:** 10.1186/1756-9966-32-86

**Published:** 2013-11-05

**Authors:** Jun Gao, Lihua Wang, Jinkang Xu, Jianming Zheng, Xiaohua Man, Hongyu Wu, Jin Jin, Kaixuan Wang, Huasheng Xiao, Shude Li, Zhaoshen Li

**Affiliations:** 1 Department of Gastroenterology, Changhai Hospital, The Second Military Medical University, Shanghai 200433, China; 2 Department of Gastroenterology, Kunshan Hospital of Traditional Chinese Medicine, Kunshan, China; 3 Department of Pathology, Changhai Hospital, The Second Military Medical University, Shanghai 200433, China; 4 Shanghai Biochip Co. Ltd, Shanghai 200433, China

**Keywords:** PDAC, DNA methyltransferases (DNMTs), Immunohistochemistry, siRNA

## Abstract

**Background:**

Altered gene methylation, regulated by DNA methyltransferases (DNMT) 1, 3a and 3b, contributes to tumorigenesis. However, the role of DNMT in pancreatic ductal adenocarcinoma (PDAC) remains unknown.

**Methods:**

Expression of DNMT 1, 3a and 3b was detected in 88 Pancreatic ductal adenocarcinoma (PDAC) and 10 normal tissue samples by immunohistochemistry. Changes in cell viability, cell cycle distribution, and apoptosis of PDAC cell lines (Panc-1 and SW1990) were assessed after transfection with DNMT1 and 3b siRNA. Levels of CDKN1A, Bcl-2 and Bax mRNA were assessed by qRT-PCR, and methylation of the Bax gene promoter was assayed by methylation-specific PCR (MSP).

**Results:**

DNMT1, 3a and 3b proteins were expressed in 46.6%, 23.9%, and 77.3% of PDAC tissues, respectively, but were not expressed in normal pancreatic tissues. There was a co-presence of DNMT3a and DNMT3b expression and an association of DNMT1 expression with alcohol consumption and poor overall survival. Moreover, knockdown of DNMT1 and DNMT3b expression significantly inhibited PDAC cell viability, decreased S-phase but increased G1-phase of the cell cycle, and induced apoptosis. Molecularly, expression of CDKN1A and Bax mRNA was upregulated, and the Bax gene promoter was demethylated. However, a synergistic effect of combined DNMT1 and 3b knockdown was not observed.

**Conclusion:**

Expression of DNMT1, 3a and 3b proteins is increased in PDAC tissues, and DNMT1 expression is associated with poor prognosis of patients. Knockdown of DNMT1 and 3b expression arrests tumor cells at the G1 phase of the cell cycle and induces apoptosis. The data suggest that DNMT knockdown may be a novel treatment strategy for PDAC.

## Introduction

Pancreatic cancer is the fourth most common cause of cancer death in the world. Pancreatic ductal adenocarcinoma (PDAC) accounts for 95% of pancreatic cancer. To date, most PDAC patients are diagnosed at advanced stages and die within 6–12 months. The overall 5-year survival rate for pancreatic adenocarcinoma is approximately 6% [1,2]. The risk factors for PDAC include cigarette smoking, heavy alcohol consumption, family history, chronic pancreatitis, and diabetes mellitus [3]. Other inconsistent risk factors include coffee, meat and fish consumption [3,4]. In addition, epidemiological evidence increasingly suggests that environmental exposures induce epigenetic modifications, altering the expression of tumor suppressor genes and oncogenes, which may be causal for pancreas tumorigenesis [5].

Altered DNA methylation, such as global hypomethylation or regional hypermethylation, is one of the most consistent epigenetic changes in human cancer. Promoter hypermethylation of tumor suppressor genes is the most common epigenetic change observed in human cancers. To date, three DNA methyltransferases (DNMTs) that are responsible for DNA methylation have been identified in mammalian cells. DNMT1 is primarily a DNA maintenance methyltransferase, as it methylates hemimethylated DNA during DNA replication *in vitro* and *in vivo*[6]. DNMT3a and DNMT3b are *de novo* methyltransferases and show similar activity on unmethylated and hemimethylated DNA [7,8]. Additionally, DNMT2 contains the full set of C-terminal catalytic domain conserved motifs, but it lacks the N-terminal regulatory domain characteristic of eukaryotic DNMT [9]. The methyltransferase activity of the recombinant DNMT2 protein is weak *in vitro* and *in vivo*[10].

Overexpression of DNMT1 3a, and 3b has been reported in a variety of cancers, such as gastric, hepatocellular, colorectal, endometrioid, prostate, breast, lung cancers, and cervical cancer [11-14]. In PDAC tissues, aberrant expression of DNMT1 and DNMT3a proteins [15-18], as well as DNMT1, 3a and 3b mRNA has been reported [19]. However, some inconsistencies were reported regarding the association with clinicopathological features and prognosis due to a small sample size. Additionally, there have been no reports regarding biological behavior alterations of PDAC cells after knockdown of DMNT expression and the underlying molecular events *in vitro*. Thus, in this study, we analyzed the expression of DNMT1, DNMT3a and DNMT3b proteins in PDAC tissue specimens and investigated their association with clinicopathological features and patient survival. Then, we knocked down DNMT expression using siRNAs to assess the effects on the regulation of biological behavior of PDAC cells and the underlying molecular mechanisms.

## Materials and methods

### Patients and tissue samples

Eighty-eight PDAC patients who had a surgically resected pancreas between January 2007 and June 2009 at the Changhai Hospital of the Second Military Medical University were recruited. None of the patients received preoperative chemotherapy or radiotherapy. Tumor and adjacent normal tissue specimens were collected in accordance with Human Subject Guidelines of the University of Washington, and this study was approved by our Institutional Review Board. The clinicopathological parameters included age, gender, smoking, alcohol consumption, serum CEA, serum CA199, location of tumor, size of tumor, nerve infiltration, metastasis of lymph nodes, tumor differentiation, and TNM staging. The post-surgery follow-up was conducted quarterly until January 2012. Tobacco smoking was defined as at least one cigarette per day for no less than one year. Alcohol consumption was defined as a one-time intake of at least 50 g of Chinese liquor, 250 ml of wine or 500 ml of beer at least once a week for a minimum of 1 year.

### Immunohistochemistry

Serial tissue sections (4 ìm thickness) were prepared from paraffin-embedded tissue blocks. The sections were deparaffinized and rehydrated, and then endogenous peroxidase activity was blocked with 3% hydrogen peroxide solution for 5 min and subjected to antigen retrieval in a microwave oven at 95°C for 12 min by immersion in antigen retrieval solution (10 mM sodium citrate buffer, pH 6.0). This was followed by a blocking step with 1% fetal calf serum for 20 min at room temperature. After that, the sections were incubated overnight at 4°C with a primary antibody including a rabbit polyclonal anti-DNMT1 antibody (1:100; Abcam, #ab19905), an anti-DNMT3a antibody (1:100; Abcam, #ab4897), or a mouse polyclonal anti-DNMT3b antibody (1:100; IMGENEX, #IMG-184A) followed by incubation with a secondary antibody (anti-mouse or anti-rabbit/ Universal Immuoperoxidase Polymer, 1:200 dilution, DAKO company, USA) at room temperature for 45 to 60 min. DAB was used for color reaction and Mayer’s hematoxylin for counterstaining. Breast and colon cancer tissue sections known for elevated DNMT1, 3a, and 3b expression were used as a positive control, as recommended by the manufacturer’s instruction. The specificity of DNMT1, 3a and 3b was verified by replacing the primary antibody with a normal serum.

Two pathologists (JZ and XM), blinded to the clinicopathological data of each section, reviewed and scored the stained sections. Nuclear staining was considered a positive stain for DNMT1, 3b, and 3a. The different staining density regions, including high, moderate, low and negative staining areas, were captured with a digital camera (Olympus U-TV0.5XC-3, 6B05290, Japan). Each region was counted for a mean of 1,000 tumor cells per case (range, 800 to 1500). According to previous studies, a section with ≥ 20% positively stained tumor cells was defined as positive protein expression [17,20]. Color intensity was not evaluated because the stains of DNMT1, 3a, and 3b in most of the samples had similar color intensity.

### PDAC cell line and culture

The poorly differentiated PDAC cell line Panc-1 and the well-differentiated cell line SW1990 were obtained from ATCC (Manassas, VA). Cells were cultured in 96-well or 6-well tissue culture plates in Dulbecco’s modified Eagle’s medium (DMEM, Invitrogen, Carlsbad, CA, USA) containing 10% fetal bovine serum (FBS, Invitrogen) at 37°C and 5% CO_2_.

### RNA isolation and quantitative-reverse transcription polymerase chain reaction (qRT-PCR)

Total RNA was isolated using a Trizol reagent (Invitrogen) and was then reversely transcribed into cDNA using a PrimeScript^TM^ RT kit (TakaRa, Dalian, China). The expression of DNMT1, DNMT3b, Bax, Bcl-2, CDKN1A, and 18S was detected by qPCR (primers are listed in Additional file 1: Table S1) in ABI7500 (Applied Biosystems, Foster City, CA, USA). The program had an initial denaturation at 95°C for 1 min followed by 40 cycles of 95°C for 15 s and 60°C for 15 s. The relative quantity (RQ) was determined using the 2^(−ÄÄCt)^ method, 18S mRNA was used as reference gene, and each experiment was repeated six or eight times.

### Construction of DNMT siRNA vectors and gene transfection

The DNMT1 and 3b-specific siRNA duplexes were chosen from the GenBank sequences (accession #NM_001379.1 and #NM_175849.1, respectively) and synthesized by Ambion Inc. (Austin, TX, USA). The DNMT1 siRNA sequences were 5′-GGAUGAGAAGAGACGUAGAtt-3′ and 5′-UCUACGUCUCUUCUCAUCCtg-3′, and the DNMT3B siRNA sequences were 5′-GCUCGUCUCCUAUCGAAAAtt-3′ and 5′-UUUUCGAUAGGAGACGAGCtt-3′. Negative control siRNA was also purchased from Ambion Inc. (Catalog #4390843). Final concentrations were 7 nM. The OptiMEM (Gibco BRL Inc.) and Lipofectamine 2000 Transfection Kits (Invitrogen) were used for siRNA transfections. Cells were plated at 1 × 10^5^ cells per well in culture dishes for overnight growth and transfected by siRNA on the following day. Six hours after transfection, the medium was removed and replaced with DMEM, and the cells were allowed to grow for 48h. Then, viability, apoptosis, cell cycle and gene expression were assayed.

### Protein extraction and western blot

Total cellular protein was extracted from cells using an M-PER mammalian protein extraction buffer (Pierce, Rockford, IL) containing 0.5 mM PMSF. The protein samples were subjected to sodium dodecyl sulfate polyacrylamide gel electrophoresis (SDS-PAGE, 12%) and electronically transferred onto PVDF membranes (Millipore, Billerica, MA, USA). The membranes were incubated with an anti-DNMT1 antibody (1:250, Abcam, Cambridge, MA, USA), an anti-DNMT3b (1:250, Imogene, Italy) or an anti-â-actin (1:5000, Sigma, St Louis, MO, USA) antibody at 4°C overnight, followed by a secondary antibody for 2 h at room temperature. The protein bands were detected with enhanced chemiluminescence (ECL) reagent (Pierce).

### Cell viability Tetrazolium Salt-8 (WST-8) assay

Briefly, cells were transfected with DNMT1, DNMT3B or negative control siRNA oligonucleotides. Seventy-two hours later, 10 ìl of WST-8 reagent was added to each well and incubated for an additional 4 h. The absorbance rate was then measured at 450 nm with an ELISA plate reader (Thermo ELISA Reader, USA).

### Flow cytometry assay

Apoptosis levels were detected using flow cytometry (Becton Dickinson) with an Annexin V-FITC Kit (Jingmei Biotech Company, Shanghai, China), while cell cycles were analyzed with propidium iodide (PI) staining.

### Methylation-specific PCR (MSP)

Genomic DNA was extracted from cultured cells using a standard proteinase K and phenol-chloroform extraction protocol. One microgram of genomic DNA was subjected to treatment with the EZ DNA Methylation Kit™ (Zymo Research, Orange, CA, USA) according to the manufacturer′s instructions. The bisulfite modified DNA was then suspended in 20 ìl of deionized water and used immediately or stored at −80°C until use. Two microliters of the bisulfite modified DNA from each sample was used as templates for methylation and unmethylation reactions. The primers of methylation-specific PCR sequences of Bax gene are shown in Additional file 1: Table S2. The PCR conditions were as follows: 95 C hot start for 5 min, then 40 repetitive cycles of denaturation (95 C for 30 s), annealing (56 C for 30 s for methylation detection, 53 C for 30 s for unmethylation detection), extension (72 C 30 s), and a final 5 min extension at 72 C. PCR products (20 ìL) were resolved on a 2% agarose gel.

### Statistical analysis

Statistical analysis was performed with SPSS version 13 (SPSS Inc., Chicago, IL). The correlation among expression of the three DNMTs was analyzed with a Kendall′s tau-b test. The correlation between the positive rate of DNMT expression and the clinicopathological characteristics of PDAC patients, as well as the difference of the variables (such as expression of DNMT1 and DNMT3b mRNA vs. tumor cell viability, apoptosis and cell cycle distribution, and expression of CDKN1A, Bcl-2, and Bax mRNA) between the differential siRNA transfected groups, were analyzed with a Mann–Whitney U test. Univariate survival analysis was performed with a Kaplan-Meier log-rank test, while multivariate survival analysis was performed with a Cox Regression test. A two-tailed P < 0.05 was considered statistically significant.

## Results

### Aberrant expression of DNMT1, 3a and 3b in PDAC tissues

**Figure 1:**
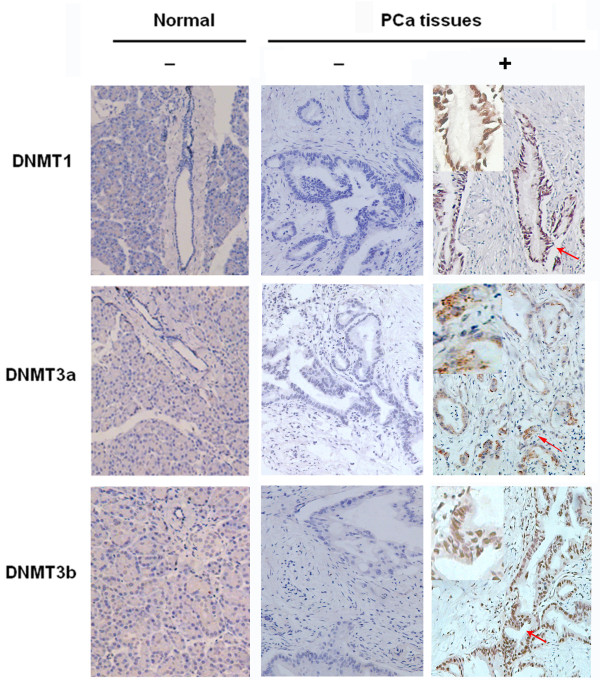
Nuclear immunostaining of DNMT1, DNMT3a and DNMT3b proteins in normal pancreatic and PDAC tissues (Original magnification: x200).

Positive staining of DNMT1, 3a, and 3b protein in PDAC tissues was observed in the cell nuclei with diffuse and/or various staining patterns by immunohistochemistry, while there was no positive staining in the adjacent normal tissues (Figure 1). The expression rates of DNMT1, DNMT3a and DNMT3b proteins were 48.9% (43/88), 23.9% (21/88) and 77.3% (68/88), respectively, in the PDAC tissue samples. In addition, DNMT3b expression significantly co-presented with DNMT3a expression (P = 0.025 with Kendall’s tau-b test), i.e., 95.2% cases that were positive for DNMT3a were also positive for DNMT3b, but not with DNMT1 expression.

### Association of DNMT1, 3a or 3b expression with clinicopathological characteristics and prognosis of the patients

**Figure 2:**
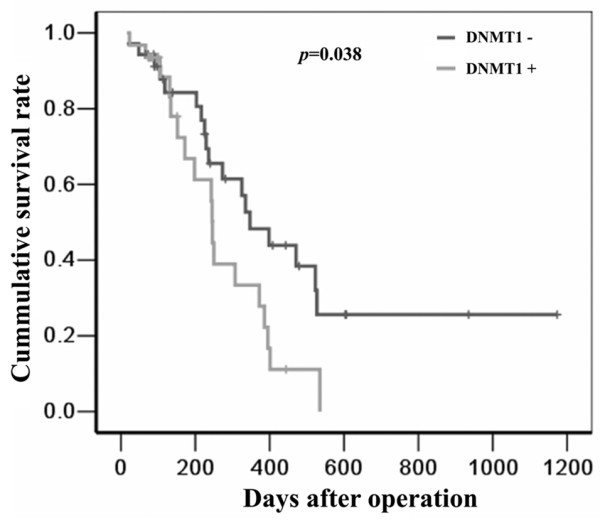
Kaplan-Meier survival curves of overall survival in PDAC patients (N = 66) according to DNMT1 expression.

The association of DNM1, 3a, and 3b expression with clinicopathological features of PDAC patients was determined. The data showed that DNMT1 expression was significantly associated with alcohol consumption, whereas the other features did not show any associations (Additional file 1: Table S3). We followed the patients for a median follow-up time of 18 months (ranging between one and 60 months), but twenty-two patients were lost the follow-up data. A univariate survival analysis was used to assess the impact of clinicopathological characteristics and DNMT1, DNMT3a and DNMT3b expression on patient survival. As shown in Figure 2, positive DNMT1 expression was significantly associated with poor overall survival (the median overall survival time: 11.6 months for positive vs. 8.2 months for negative), a high TNM stage, poor differentiation, lymph node metastasis and serum CEA (Additional file 1: Table S4). The multivariate survival analysis, in which the significant variants from the univariate survival analysis were inputted into the Cox regression model, identified DNMT1 positive expression, poor differentiation and serum CEA as independent prognostic factors for poor overall survival (Additional file 1: Table S5).

### Effects of DNMT1 and DNMT3b siRNA on regulation of PDAC cell viability, apoptosis, and cell cycle distribution

**Figure 3:**
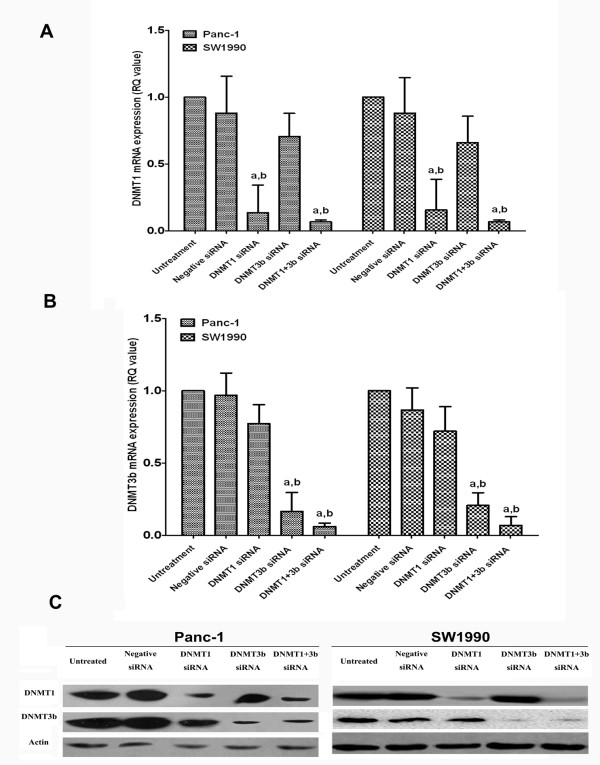
**Knockdown of DNMT1 and DNMT3b expression in PDAC cells.** DNMT1 siRNA, DNMT3b siRNA, or their combination was transiently transfected into PDAC cell lines and then subjected to qRT-PCR **(A,B)** and western blotting **(C)** analysis for DNMT1 and DNMT3b expression. a, p < 0.05 compared with the untreated cells. b, p < 0.05 compared with the negative siRNA cells. The data are shown as the mean ± SD of six independent experiments.

**Figure 4:**
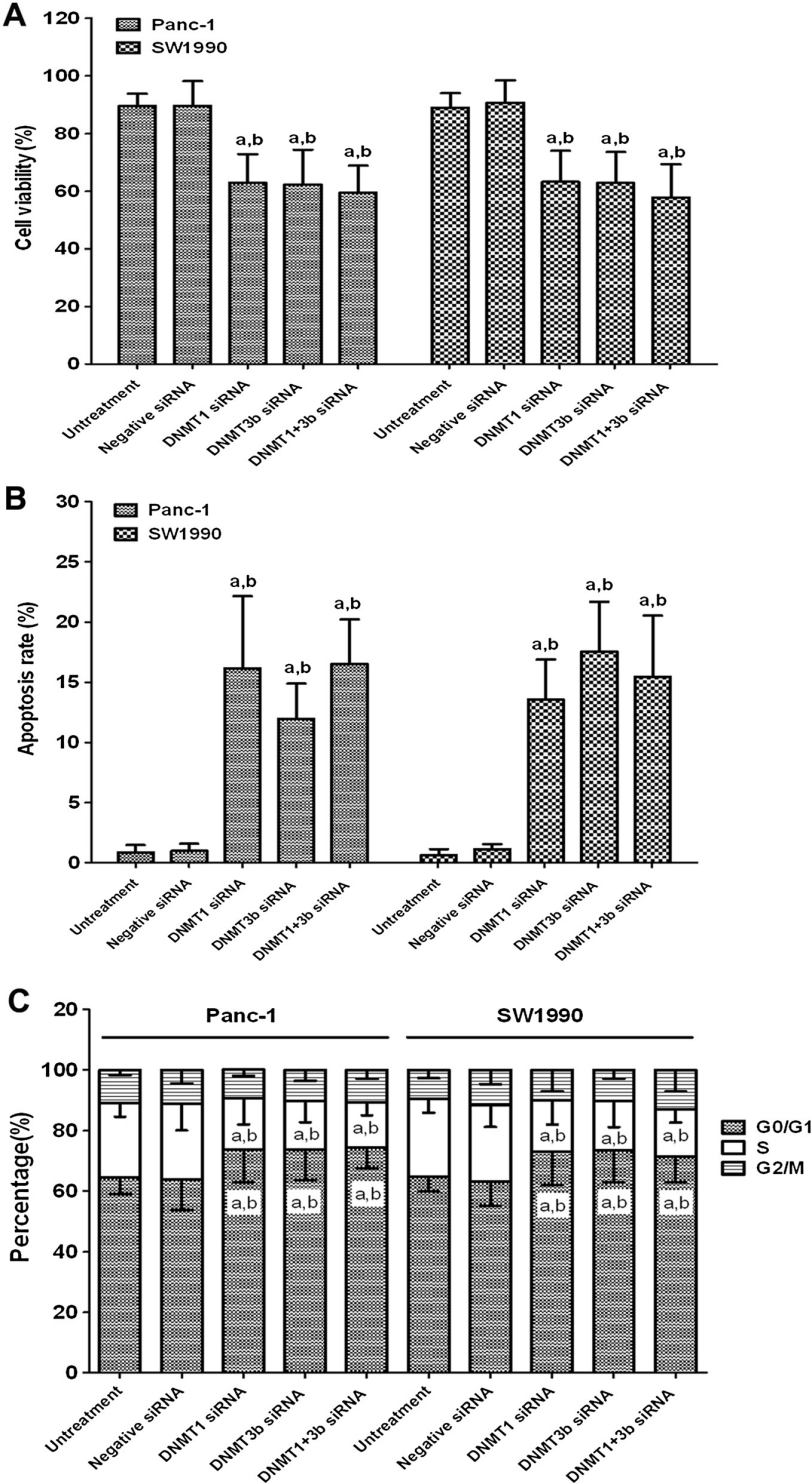
**Effects of DNMT1 and DNMT3b siRNA on cell viability (A), apoptosis (B) and cell cycle distribution (C).** a, p < 0.05 compared with the untreated cells. b, p < 0.05 compared with the negative siRNA-transfected cells. The data are shown as the mean ± SD of eight independent experiments.

The rates of aberrant DNMT1 and DNMT3b expression were much higher than that of DNMT3a in PDAC, and 95.2% of the cases positive for DNMT3a were also positive for DNMT3b. Thus, we knocked down DNMT1 and 3b. It is possible that DNMT3a could also play a role in PDAC progression. PDAC cell lines Panc-1 (poorly differentiated) and SW1990 (well-differentiated) were assessed. As shown in Figure 3, both qRT-PCR and Western blot data showed that transfection of DNMT1 and DNMT3b siRNA significantly reduced expression of their corresponding mRNA and protein. Transfection of either DNMT1 or DNMT3b siRNA significantly inhibited the viability of PDAC cells and decreased the number of cells in the S-phase, increased the number of cells in the G0/G1-phase, and induced apoptosis, with no significant difference between DNMT1 and DNM3b (Figure 4). Combination of DNMT1 or DNMT3b siRNA did not have any synergistic effects.

### Effect of DNMT1 and DNMT3b siRNA on the modulation of CDKN1A, Bcl-2, and Bax expression

**Figure 5:**
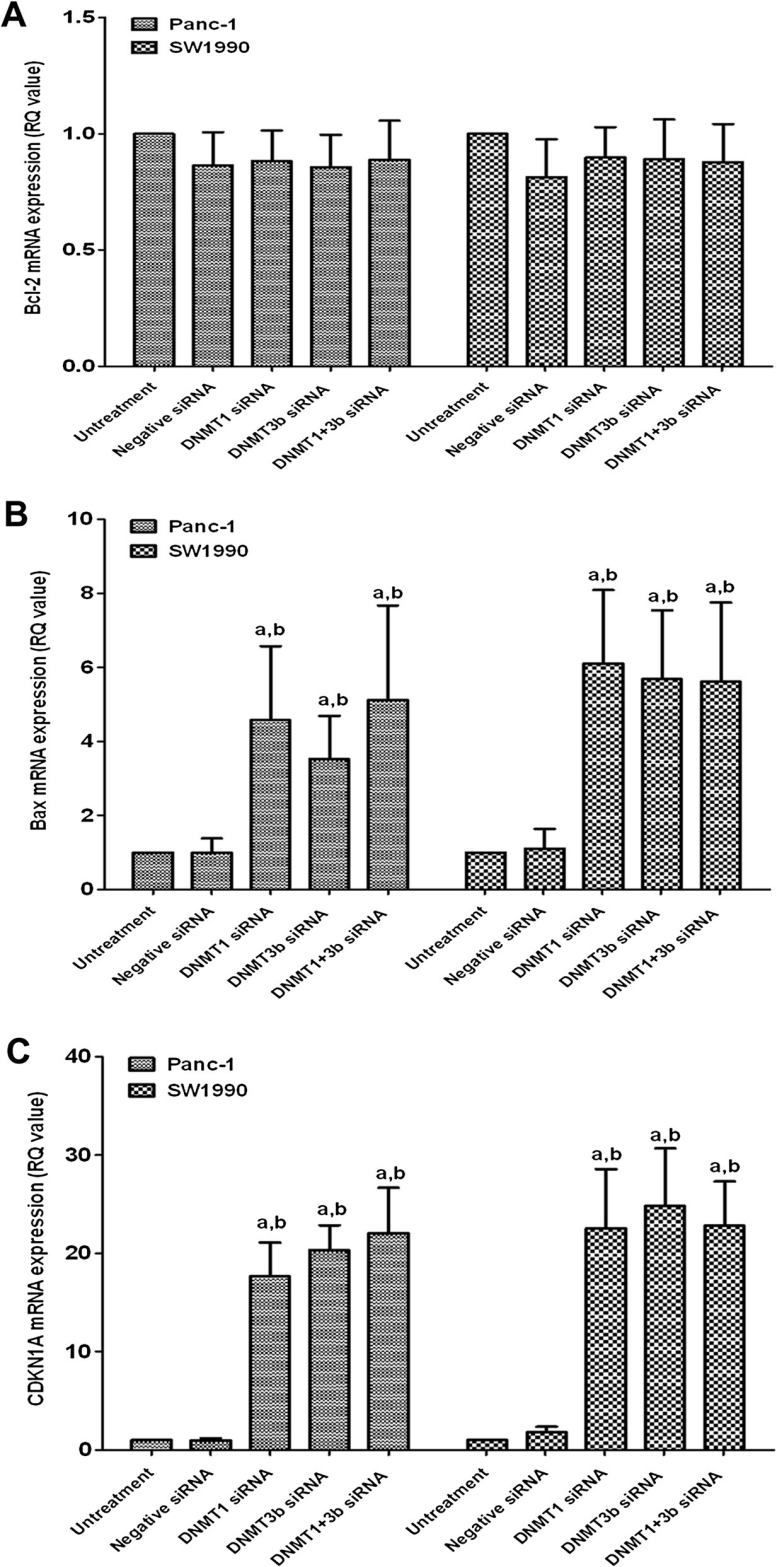
**Effects of DNMT1 and DNMT3b siRNA on the regulation of Bcl-2 (A), Bax (B) and CDKN1A (C) expression.** a, p < 0.05 compared with the untreated cells. b, p < 0.05 compared with the negative siRNA-transfected cells. The data are shown as the mean SD of six independent experiments.

**Figure 6:**
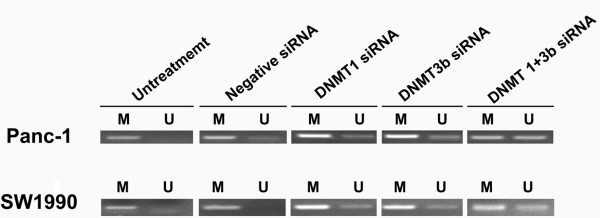
**Demethylation of Bax gene promoter was determined by MSP method after DNMT1 siRNA, DNMT3b siRNA, or their combination was transiently transfected into PDAC cell lines.** M, Methylation. U, Un-methylation.

The expression of the cell cycle-related molecule CDKN1A and the apoptosis-related molecule Bcl-2 and Bax was detected by qRT-PCR. As shown in Figure 5, expression of CDKN1A and Bax mRNA was significantly upregulated in the DNMT1 siRNA, DNMT3b siRNA, and DNMT1 + DNMT3b siRNA groups, whereas Bcl-2 mRNA was not significantly changed in all groups. However, the combination of DNMT1 and DNMT3b siRNA did not have a synergistic effect. Additionally, the methylation status of the Bax promoter was determined by methylation-specific PCR (MSP). The demethylation effects of either DNMT1 or DNMT3b siRNA or their combination are shown in Figure 6. These results suggest that upregulated Bax mRNA expression may be due to DNA demethylation.

## Discussion

In the current study, we showed that the altered expression of DNMT1, 3a, and 3b was associated with PDAC tumorigenesis and progression. Specifically, aberrant expression of DNMT1, 3a and 3b proteins was found in PDAC tissues, and DNMT1 expression was significantly associated with alcohol consumption and poor survival of PDAC patients. Knockdown of DNMT1 and DNMT3b expression significantly inhibited the viability of PDAC cells, decreased the number of cells in the S-phase, increased the number of cells in the G0/G1-phase, induced apoptosis, upregulated the mRNA expression of the cell cycle-related CDKN1A gene and the pro-apoptotic Bax gene, and demethylated Bax promoter DNA. Although a synergistic effect of DNMT1 and DNMT3b siRNA knockdown was not observed, DNMT silencing may be a potential novel treatment strategy for PDAC.

Previous studies have demonstrated that hypermethylation of tumor suppressor gene promoters is crucial for cancer initiation and progression [21]. Many CpG islands in the gene promoters have been reported to be hypermethylated in PDAC [22], such as SPARC [23], CDKN2A(p16) [24], RASSF1A [25], WWOX [25] and CXCR4 [26]. It has been speculated that the aberrant hypermethylation of tumor suppressor genes could be caused by abnormal upregulation of DNMT expression. In previous studies, it was shown that the overexpression of DNMT1 [16,17] and DNMT3a [26] in PDAC tissues was associated with poor overall survival, but the association data were inconsistent. In the current study, expression of DNMT1, 3a and 3b proteins was simultaneously analyzed in PDAC tissues and a more detailed investigation of clinicopathological characteristics was performed. A comprehensive knowledge about the pathogenic characteristics of DNMTs involved in PDAC tumorigenesis was gathered and our data indicate that overexpression of DNMT1 and DNMT3b play a key role in PDAC tumorigenesis and progression.

Hypermethylation of tumor suppressor genes is caused by aberrant up-regulation of DNMT protein expression and enzyme activity. Thus, many researchers have targeted DNTM as a novel treatment strategy for tumors In addition to cytosine analogues capable of trapping DNMTs onto DNA, inhibition of DNMT mRNA expression through siRNA [15,27], antisense [15], irradiation [28,29] and mediation through the Hedgehog pathway [18] have been explored in previous studies. Some studies have also investigated the synergistic knockdown of DNMT1 and DNMT3b [27,30,31]. In fact, our data did not show a synergistic effect after combination of DNMT1 and DNMT3b siRNA in PDAC cells. These results were in agreement with previous studies in breast [31] and PDAC cell lines [27], as well as in lung, esophageal cancer, and malignant pleural mesothelioma cells [32]. In contrast, other studies have shown a synergistic effect in an ovarian cancer CP70 cell line [33], a cholangiocarcinoma QBC-939 cell line [31] and a colorectal cancer cell line [34]. We speculated that this discrepancy might be attributed to different hypermethylation mechanisms in the different types of cancers. For example, PDAC may have its own gene methylation profile during tumorigenesis and progression [35,36]. Thus, further *in vivo* studies are needed to confirm whether there is a synergistic effect after combination of DNMT1 and DNMT3b siRNA in PDAC.

As stated, DNMT1 and DNMT3b siRNA transfection reduced PDAC cell viability, arrested cells at the G0/G1-phases, decreased the number of cells at the S-phase of the cell cycle and induced apoptosis of PDAC cells. In order to explore the associated mechanism, we analyzed the expression of Bcl-2 and Bax mRNA [37]. In this study, Bax mRNA was significantly upregulated, while Bcl-2 mRNA was not altered. This may have been due to demethylation of the Bax gene promoter after DNMT1 and DNMT3b siRNA transfection. This finding is in agreement with the hypothesis that Bax may be more vulnerable to environmental factors than Bcl-2 in PDAC [38]. In addition, the expression of CDKN1A mRNA was analyzed because CDKN1A mediates cell cycle arrest in response to the p53 checkpoint pathway during PDAC tumorigenesis [39-41]. CDKN1A mRNA was significantly upregulated after DNMT1 and DNMT3b siRNA transfection. The results were consistent with previous studies showing that DNMT inhibition led to a rapid upregulation of CDKN1A expression [42].

The results of the present study demonstrated that DNMT1, 3a and 3b proteins were highly expressed in PDAC tissues, suggesting that DNMTs may be targets for carcinogenic environmental factors to affect tumor suppressor gene methylation. Knockdown of DNMT expression is a potential strategy for PDAC treatment.

## Competing interests

There are no competing interests to declare.

## Authors’ contributions

SDL, ZSL and JG designed the study. LHW and JG wrote the manuscript. JKX and HXM collected the samples. JMZ and HXM performed the immunohistochemistry and scoring. JJ and KXW performed the qRT-PCR analysis. HYW carried out cell culture. JG, LHW and JKX analyzed data. All authors read and approved the final manuscript.

## Supplementary Material

Additional file 1 **Table S1.** Primers for Real-time RT-PCR. **Table S2.** Methylation-specific PCR primers for Bax gene promoter.**Table S3.** Association of DNMT1, 3a, or 3b expression with clinicopathological characteristics for PDAC patients. **Table S4.** Univariate survival analysis (Log Rank) of the clinicopathological characteristics of PDAC patients. **Table S5.** Multivariate survival analysis (Cox regression) of the clinicopathological characteristics of PDAC patients. Click here for file
