# Supplementary material for: Aberrant DNA methyltransferase expression in pancreatic ductal adenocarcinoma development and progression
Source: J Exp Clin Cancer Res. 2013 Nov 5;32(1):86. doi: 10.1186/1756-9966-32-86 (PMC4029463; doi:10.1186/1756-9966-32-86)
Supplement: Additional file 1 — Table S1. Primers for Real-time RT-PCR. Table S2. Methylation-specific PCR primers for Bax gene promoter.Table S3. Association of DNMT1, 3a, or 3b expression with clinicopathological characteristics for PDAC patients. Table S4. Univariate survival analysis (Log Rank) of the clinicopathological characteristics of PDAC patients. Table S5. Multivariate survival analysis (Cox regression) of the clinicopathological characteristics of PDAC patients. [file 1756-9966-32-86-S1.doc]

**Supplemental Table 1.** Primers for Real-time RT-PCR.

| **Gene** | **Sense** | **Antisense** | **Size (bp)** |
| --- | --- | --- | --- |
| DNMT1 | 5'-GTGGGGGACTGTGTCTCTGT-3' | 5'-TGAAAGCTGCATGTCCTCAC-3' | 204 |
| DNMT3B | 5'-TTGAATATGAAGCCCCCAAG-3' | 5'-TGATATTCCCCTCGTGCTTC-3' | 201 |
| Bcl-2 | 5'-TCCATGTCTTTGGACAACCA-3' | 5'-CTCCACCAGTGTTCCCATCT-3' | 203 |
| Bax | 5'-TGTCTGTCTTGTCCCCTTCC-3' | 5'-ACCTTGAGCACCAGTTTGCT-3' | 301 |
| CDKN1A | 5'-TTAGCAGCGGAACAAGGAGT-3' | 5'-AGAAACGGGAACCAGGACAC-3' | 252 |
| 18S | 5'-CTCTTAGCTGAGTGTCCCGC-3' | 5'-CTGATCGTCTTCGAACCTCC-3' | 294 |

**Supplemental Table 2**. Methylation-specific PCR primers for Bax gene promoter.

| **Gene** | **Up primer** | **Down primer** | **Size (bp)** |
| --- | --- | --- | --- |
| Bax Methylation | 5'- ACGTGACGGGATTAAATTTTTC -3 | 5'- AAAAACCCCGCTAAACGT -3' | 124 bp |
| Bax Unmethylation | 5'- GTTATGTGATGGGATTAAATTTTTT -3 | 5'-AAAAACCCCACTAAACATACA -3' | 124 bp |

**Supplemental Table 3.** Association of DNMT1, 3a, or 3b expression with clinicopathological characteristics for PCa patients.

| Variable | DNMT1 | | | DNMT3A | | | | DNMT3B | | | |
| --- | --- | --- | --- | --- | --- | --- | --- | --- | --- | --- | --- |
|  | Total | Positive (%) | *P* | | Total | Positive (%) | *P* | | Total | Positive (%) | *P* |
| Age |  |  | 0.996 | |  |  | 0.896 | |  |  | 0.055 |
| ＜60 | 45 | 22 (48.9) |  | | 45 | 11 (24.4) |  | | 45 | 31(68.9) |  |
| ≥60 | 43 | 21 (48.8) |  | | 43 | 10 (23.3) |  | | 43 | 37(86.0) |  |
| Sex |  |  | 0.409 | |  |  | 0.949 | |  |  | 0.431 |
| Male | 55 | 25(45.5) |  | | 55 | 13(23.6) |  | | 55 | 44(80.0) |  |
| Female | 33 | 18(54.5) |  | | 33 | 8 (24.2) |  | | 33 | 24(72.7) |  |
| Tobacco smoking |  |  | 0.311 | |  |  | 0.434 | |  |  | 0.634 |
| Yes | 27 | 11(40.7) |  | | 27 | 5 (18.5) |  | | 27 | 20 (74.1) |  |
| No | 61 | 32 (52.5) |  | | 61 | 16 (26.2) |  | | 61 | 48(78.7) |  |
| Alcohol consumption |  |  | 0.001 | |  |  | 0.126 | |  |  | 0.755 |
| Yes | 24 | 5 (20.8) |  | | 24 | 3(12.5) |  | | 24 | 18(75.0) |  |
| No | 64 | 38 (59.4) |  | | 64 | 18(28.1) |  | | 64 | 50 (78.1) |  |
| Serum CEA |  |  | 0.673 | |  |  | 0.896 | |  |  | 0.694 |
| <5ng/mL | 45 | 21(46.7) |  | | 45 | 11(24.4) |  | | 45 | 34 (75.6) |  |
| ≥5ng/mL | 43 | 22(51.2) |  | | 43 | 10 (23.3) |  | | 43 | 34 (79.1) |  |
| Serum CA199 |  |  | 0.896 | |  |  | 0.475 | |  |  | 0.795 |
| <37U/mL | 24 | 12(50.0) |  | | 24 | 7 (29.2) |  | | 24 | 19 (79.2) |  |
| ≥37U/mL | 64 | 31(48.4) |  | | 64 | 14 (21.9) |  | | 64 | 49 (76.6) |  |
| Tumor location |  |  | 0.408 | |  |  | 0.741 | |  |  | 0.981 |
| Head | 53 | 24 (45.3) |  | | 53 | 12 (22.6) |  | | 53 | 41 (77.4) |  |
| Body and tail | 35 | 19 (54.3) |  | | 35 | 9 (25.7) |  | | 35 | 27 (77.1) |  |
| Tumor size |  |  | 0.408 | |  |  | 0.087 | |  |  | 0.587 |
| ≤3 | 35 | 19 (54.3) |  | | 35 | 5 (14.3) |  | | 35 | 26(74.3) |  |
| ＞3 | 53 | 24 (45.3) |  | | 53 | 16 (30.2) |  | | 53 | 42 (79.2) |  |
| Nerve infiltration |  |  | 0.207 | |  |  | 0.553 | |  |  | 0.833 |
| No | 37 | 21 (56.8) |  | | 37 | 10 (27.0) |  | | 37 | 29 (78.4) |  |
| Yes | 51 | 22 (43.1) |  | | 51 | 11 (21.6) |  | | 51 | 39 (76.5) |  |
| Lymph node metastasis |  |  | 0.056 | |  |  | 0.991 | |  |  | 0.459 |
| No | 42 | 25 (59.5) |  | | 42 | 10 (23.8) |  | | 42 | 31(73.8) |  |
| Yes | 46 | 18 (39.1) |  | | 46 | 11 (23.9) |  | | 46 | 37 (80.4) |  |
| Tumor differentiation |  |  | 0.525 | |  |  | 0.977 | |  |  | 0.152 |
| Poor | 37 | 17 (45.9) |  | | 37 | 9 (24.3) |  | | 37 | 25(67.6) |  |
| Moderate | 20 | 12 (60.0) |  | | 20 | 5 (25.0) |  | | 20 | 16(80.0) |  |
| Well | 31 | 14 (45.2) |  | | 31 | 7 (22.6) |  | | 31 | 27(87.1) |  |
| TNM staging |  |  | 0.090 | |  |  | 0.727 | |  |  | 0.944 |
| I+II | 39 | 23 (59.0) |  | | 39 | 10 (25.6) |  | | 39 | 30(76.9) |  |
| III+IV | 49 | 20 (40.8) |  | | 49 | 11 (22.4) |  | | 49 | 38 (77.6) |  |

**Supplemental Table 4. Univariate survival analysis (Log Rank) of the clinicopathological characteristics of PCa patients.**

| **clinicopathological characteristics** | Cases | Events | Median survival (months) | p value |
| --- | --- | --- | --- | --- |
| Age |  |  |  | 0.848 |
| <60 | 35 | 21 | 11.56 |  |
| ≥60 | 31 | 15 | 8.20 |  |
| Sex |  |  |  | 0.185 |
| Male | 41 | 24 | 8.16 |  |
| Female | 25 | 12 | 11.56 |  |
| Tobacco smoking |  |  |  | 0.686 |
| No | 46 | 22 | 10.23 |  |
| Yes | 20 | 14 | 10.83 |  |
| Alcohol consumption |  |  |  | 0.981 |
| No | 49 | 25 | 10.23 |  |
| Yes | 17 | 11 | 10.83 |  |
| Serum CEA |  |  |  | 0.022 |
| <5ng/mL | 35 | 20 | 12.40 |  |
| ≥5ng/mL | 31 | 16 | 8.16 |  |
| Serum CA199 |  |  |  | 0.760 |
| <37U/mL | 18 | 8 | 9.10 |  |
| ≥37U/mL | 48 | 28 | 13.16 |  |
| Tumor location |  |  |  | 0.971 |
| Head | 50 | 29 | 10.23 |  |
| Body and tail | 16 | 7 | 13.16 |  |
| Tumor size |  |  |  | 0.693 |
| ≤3 | 26 | 14 | 11.16 |  |
| ＞3 | 40 | 22 | 9.10 |  |
| Nerve infiltration |  |  |  | 0.777 |
| No | 28 | 16 | 11.16 |  |
| Yes | 38 | 20 | 9.10 |  |
| Lymph node metastasis |  |  |  | 0.035 |
| No | 31 | 12 | 7.20 |  |
| Yes | 35 | 24 | 8.16 |  |
| Tumor differentiation |  |  |  | 0.005 |
| Poor | 26 | 13 | 7.20 |  |
| Moderate | 14 | 5 | 12.40 |  |
| Well | 26 | 18 | 13.26 |  |
| TNM staging |  |  |  | 0.043 |
| I+II | 30 | 11 | 12.40 |  |
| III+IV | 36 | 25 | 8.10 |  |
| DNMT1 expression |  |  |  | 0.038 |
| Negative | 35 | 18 | 11.56 |  |
| Positive | 31 | 18 | 8.20 |  |
| DNMT3A expression |  |  |  | 0.820 |
| Negative | 50 | 29 | 10.23 |  |
| Positive | 16 | 7 | 9.10 |  |
| DNMT3B expression |  |  |  | 0.325 |
| Negative | 15 | 8 | 5.06 |  |
| Positive | 51 | 28 | 11.16 |  |

**Supplemental Table 5.** Multivariate survival analysis (Cox regression) of the clinicopathological characteristics of PCa patients.

|  | Overall survival | |
| --- | --- | --- |
|  | RR (95% CI) | p value |
| Lymph metastasis |  | 0.837 |
| No | 1 |  |
| Yes | 1.253(0.146-10.779) |  |
| TNM staging |  | 0.750 |
| I+II | 1 |  |
| III+IV | 1.436(0.155-13.272) |  |
| Tumor differentiation |  | 0.012 |
| Well | 1 |  |
| Moderate | 1.484(0.512-4.302) | 0.107 |
| Poor | 3.685(1.515-8.963) | 0.004 |
| Serum CEA |  | 0.017 |
| <5ng/mL | 1 |  |
| ≥5ng/mL | 2.444(1.175-5.084) |  |
| DNMT1 expression |  | 0.029 |
| Negative | 1 |  |
| Positive | 2.107 (1.078-4.116) |  |
